# Statin Treatment Induced a Lipogenic Expression Hierarchical Network Centered by SREBF2 in the Liver

**DOI:** 10.3389/fendo.2021.573824

**Published:** 2021-07-19

**Authors:** Shiyu Song, Mengyuan Niu, Qiao Liang, Lei Wang, Haiyan Min, Yuming Peng, Hongwei Wang, Qian Gao

**Affiliations:** ^1^ Center for Translational Medicine and Jiangsu Key Laboratory of Molecular Medicine, Medical School of Nanjing University, Nanjing, China; ^2^ Department of General Practice of Central Hospital of Karamay, Karamay, China; ^3^ State Key Laboratory of Analytical Chemistry for Life Science, Medical School, Nanjing University, Nanjing, China

**Keywords:** statin, liver, protein acetylation, WGCNA, obesity, diabetes

## Abstract

Statin treatment is a major prevention treatment for hypercholesterolemia and the management of patients with increased risk of cardiovascular disease (CVD) due to its protective effects. However, its long-term safety was questioned regarding its potential role in new-onset type 2 diabetes mellitus, and its effect on gene regulation in the liver is not yet fully understood. By reanalyzing the transcriptome of the livers of patients with obesity and hypercholesterolemia, this study shows a multiple module organization that is related to various clinical metabolic parameters and identified an expression hierarchical network involving cholesterol and fatty acid syntheses in the liver of statin-treated patients. The key genes of the network were validated by QPCR in the hepatocytes upon statin treatment. The upregulation of the key enzymes involving the synthesis of Acetyl-CoA and the induction of gentle global acetylation of pan-protein and histone H4 in hepatocytes were observed. The study provides an overall view of the statin effect on transcriptional and post-transcriptional regulation of genes in the liver.

## Introduction

The high prevalence of obesity is a worldwide health challenge that has continually increased in the last 40 years. It is believed that the global rates of overweight people have almost tripled since 1975 to approximately 39% (1.9 billion) of grown-ups, more than 13% (650 million) of whom are obese (1, 2). Obesity is associated with multiple metabolic disorders, e.g. type 2 diabetes, hypertension, dyslipidemia, cardiovascular disease (CVD), NASH, and many types of cancers. Among them, CVD is becoming the most common cause of death, and total figures have now surpassed 17.3 million deaths a year globally, more than twice that caused by cancers according to the Global Burden of Disease (GBD) study (3).

There is overwhelming evidence on the benefits of reducing low-density lipoprotein cholesterol (LDL-c) by statin, a 3-hydroxy-3-methylglutaryl-coenzyme A (HMG-CoA) reductase inhibitor, to CVD (4). Although the protective effect of statin on CVD is supported by multiple clinical evidence, its long-term safety was questioned. For example, several randomized controlled trials (RCTs) and observational studies have outlined the possibility of new-onset type 2 diabetes mellitus with statin treatment, especially in those who have preexisting risk in glucose metabolism (4, 5). However, little is known about the overall response of the body or organs to statin treatment.

The liver is a central organ in lipogenesis, gluconeogenesis, cholesterol metabolism, and is the major metabolic organ for statin. Thus, the effects of statin on the liver, for example on its gene regulation, should be addressed. Previously, differently expressed gene (DEGs) analysis in the livers between statin treated and not treated patients was performed (6); however, the differences of the DEGs were small and the noises of transcription were high. The overall organization of the altered gene modules was not accessed.

Weighted gene correlation network analysis (WGCNA) is a powerful method for constructing co-expression networks based on expression data with an unbiased approach (7). It provides a system biology strategy to explore the functionality of a transcriptome at organ-/system-level that is especially suitable for analyzing the correlations of corresponding module eigengene with numeric clinical phenotypes to identify key modules/genes in a transcriptome.

The present study performed a WGCNA to analyze the transcriptome of a microarray data set of liver samples of 910 severely obese patients with/without statin treatment. Various clinical parameters were correlated with organized gene modules. 4 modules were correlated with clinical metabolic phenotypes and recognized among a total of 18 modules. One module, the light green module with 75 genes, showed stronger correlations with glucose and cholesterol parameters and statin treatment. Gene enrichment and interaction analysis revealed that 19 biologically tightly associated genes form a gene hub that is significantly correlated with low plasma cholesterol and high fasting glucose levels of the patients to the statin treatment. This gene expression network was centered by the transcriptional factor SREBF2 with several novel key genes that were previously not identified by the DEG analysis. Finally, the transcriptional upregulation of key genes was validated by QPCR in hepatocytes. Enhanced acetylation of both global and histone was observed after statin treatment. Our findings explored an integrated transcriptional response and post-transcriptional alternation of the liver by statin.

## Material and Method

### Data Collecting and Processing

Dataset GSE130991, a comprehensive analysis of the liver transcriptome of obese patients who participated in the Biological Atlas of Severe Obesity (ABOS) cohort (ClinicalTrials.gov identifier NCT01129297) was initially downloaded from GEO via GEOquery (8). The raw data were previously processed with robust multi-array average (RMA) in log2 transformed form, was then normalized by limma R package (9). The gene symbols were mapped to the probe ID according to the annotation of GPL20265 [HTA-2_0] Affymetrix Human Transcriptome Array 2.0 [transcript (gene) version]. Probes that do not match with mature transcription were removed. Genes matching multiple probes, the highest average expression of the probe was chosen. Finally, 25,128 genes were identified to undergo WGCNA analysis.

The clinical factor data such as gender and usage of statin were transformed to numeric data before proceeding. The cluster and correlation test of the clinical parameters were based on K‐means algorithm with Spearman distance and COR function with Spearman’s method, respectively. Principal component analysis (PCA) was applied to show the overall profile of patients at the transcriptional level.

### Weighted Gene Co-expression Network Construction and Module Detection

WGCNA was carried out on all 25,128 genes using the R “WGCNA” package. First, cluster analysis was performed by flashClust to evaluate if there was an obvious outlier among the samples. Among the 910 objects, 876 were kept for network construction. Next, an appropriate soft thresholding power β of 12 for network construction was identified with the function Pick Soft Threshold in the ‘WGCNA’ package to avoid the selection of an arbitrary cut-off. Co-expression similarity for each pair of genes from the adjacency matrix is determined via the Topological Overlap Matrix (TOM) method which could minimize the effects of noise and spurious associations. Finally, the co-expression gene modules were identified by hierarchical cluster analysis with the following major parameters: maxBlockSize of 20,000, minModuleSize of 50, and deepSplit of 2.18 modules were obtained after merging highly similar modules with the height of module eigengene (ME) calculated by retaining the first principal component following principal component analysis.

### Relating Modules to Clinical Parameters and Discovering Modules of Interest

The relevance between MEs and clinical parameters was calculated by COR function with Spearman’s method in the WGCNA. The log10 transformation of the P value was then defined as gene significance (GS) and the average GS for all genes in the module was defined as the module significance (MS).

### Functional Enrichment Analysis

Gene ontology (GO) biological process enrichment and KEGG pathway enrichment of genes in the target module were analyzed and visualized by the “clusterProfiler” R package (10).

### Identification and Validation of Hub Genes

The gene correlation network of the interested module was calculated with the TOM method. For biological interactions, the genes were submitted to Search Tool for the Retrieval of Interacting Genes (STRING, http://www.string-db.org/) (11), and the protein–protein interaction (PPI) networks were retrieved. The PPI network was analyzed and visualized by Cytoscape v3.7.2 software (12). The nod size was mapped according to the average shortest path and the width of the edges was mapped according to the combined score, indicating the strength of interaction between the nods according to TOM. The expressions of the feature genes of the hub were then analyzed by Spearman Ranked Assay between statin treated or not treated group. The correlation with clinical parameters is analyzed by COR function in R with Spearman’s method (13).

### Cell Culture and Treatment

The human hepatocyte cell line LO2 was purchased from the Type Culture Collection of the Chinese Academy of Sciences (Shanghai, China). The cells were grown in Dulbecco’s modified Eagle’s medium (DMEM) supplemented with 10% (v/v) fetal bovine serum (FBS) and 1% penicillin streptomycin (Gibco, USA) in humidified incubators at 37°C under 5% CO_2_. The cells were seeded in 6-well plates and treated with the indicated concentration of lovastatin, simvastatin, or TSA (Selleck, USA) for 24 hours.

### Cell Viability Assay

Cells were seeded in triplicate at a density of 2 ×10^4^ cells/mL in 96 well plates, and Cell viability assays were performed using the CellTiter 96 AQueous One Solution Cell Proliferation Assay kit (Promega, USA) after treatment with 2-fold diluted statins for 24 hours. The absorbance at 490 nm was measured using a microplate reader (Bioteck, USA).

### RNA Extraction and QPCR

mRNA was extracted from cultured cells using RNeasy Micro Kit (Qiagen, Germany), Total mRNA was reversed transcribed into cDNA with PrimeScript RT Master Mix (TaKaRa, otsu, Japan). SYBR green quantitative real-time was performed, using PCR Master Mix (Life technology, USA). The expression of the target gene was determined relative to beta actin and relative expression was calculated by ΔΔCt method. The primers of each gene are listed in **Table S1**.

### Protein Extraction and Western Blot

Cell total proteins were extracted using RIPA buffer and the protein concentrations were determined by a BCA kit (Thermo, USA). 20 μg of total lysates were separated by a 10% SDS-PAGE gel and transferred onto a PVDF membrane and blotted with 5% bovine serum albumin (BSA) in TBS for 90 minutes, and then incubated with primary antibodies of anti-acetyl-lysine, anti-acetyl histone H4, and anti-total histone H4 (Cell Signaling Technology, USA) overnight at 4°C. After washing, the membrane was incubated with HRP conjugated horse anti-rabbit antibody, 60 minutes at room temperature. The membranes were visualized by ECL Plus western blotting detection reagents (Millipore, USA). Total histone H4 was used as an internal control. The gel was stained with Coomassie Brilliant Blue G250.

## Results

### Identification of Main Clinical Parameters Correlated With Statin Treatment

**Figure 1:**
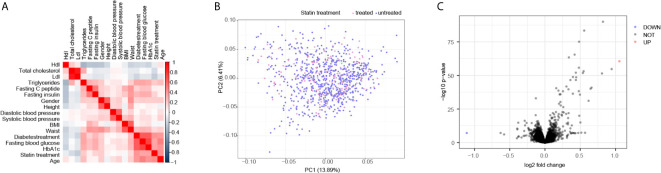
Correlated test identified main clinical parameters with Statin treatment. **(A)** Correlation matrix according to Spearman’s rank correlation coefficient of 17 clinical parameters showed the positive correlation of glucose metabolic and negative correlation of cholesterol with statin treatment. **(B)** Scatter plot of Principal component analysis (PCA) according to gene transcription levels, the color of the dots was mapped to the condition of statin treatment of the patients. **(C)** Volcano plots of the DEGs are depicted with the fold change of each gene and the p value was calculated by performing a Welch’s t-test. The genes whose absolute value of Log2 fold change > 1 (2 folds) and p value < 0.05 were considered as significantly changed genes. Only two genes were significantly changed, one up in red and one down in blue, according to this criterion.

To identify the clinical observations that correlated with statin treatment, we initially compared a set of 17 available measurements (**Figure S1**) that were collected from a cohort of 910 overweight patients and performed Spearman’s correlation test. As shown in **Figure 1A**, the statin treatment was positively correlated with fasting blood glucose and HbA1c, but reversely correlated with total cholesterol and Ldl, suggesting a beneficial role of statin on cholesterol metabolism, but a potential risk on glucose metabolism. However, the scatter‐plot based on PCA of the liver transcriptome data did not show separated clusters according to the treatment of statin, indicating that the overall profiles of the transcriptome were not significantly altered by statin treatment in the liver (**Figure 1B**). Moreover, the DEGs analysis showed that there were seldom genes in the liver that varied more than 2 fold between statin treated and non-treated patients (**Figure 1C**). Together, these results indicated obvious alterations in clinical measurements regarding the cholesterol and glucose metabolism by statin treatment, but minimum gene alternations at the transcription level. Thus we were encouraged to investigate the association of the internal expression organization of the genes in responding to statin treatment to reveal subtle but significant differentially expressed genes through their concurrent expression behavior.

### Construction of Weighted Gene Correlation Network Revealed 18 Gene Modules

**Figure 2:**
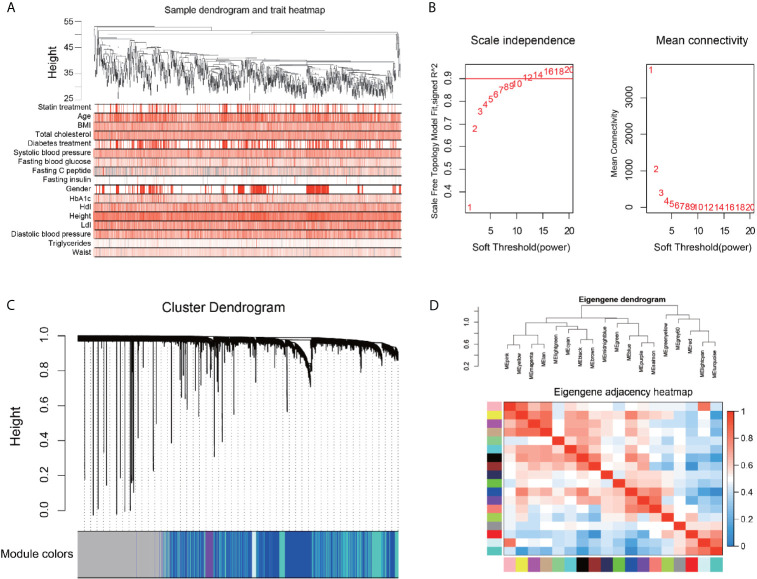
Construction of gene correlation network by Weighted gene co-expression network analysis. **(A)** Clustering dendrogram of 876 samples and the clinical traits. The clustering was based on the expression data of the liver. The color intensity was proportional to higher levels of the indicated parameter. **(B)** Network topology for different soft-thresholding powers. Numbers in the plots represent the corresponding soft thresholding powers, and the approximate scale-free topology can be acquired at the soft-thresholding power of 12. **(C)** Gene dendrogram is based on the same topological overlap with the corresponding color row-indicated module colors. A total of 18 modules were identified. The genes which are not coexpressed in any modules are assigned to the gray module. **(D)** Hierarchical clustering dendrogram (upper panel) and the correlation heatmap (lower panel) of each module are according to module eigengenes. Different colors of the abscissa and the ordinate represent different modules.

We performed an unbiased WGCNA analysis with all annotated genes obtained from the liver samples of the patients. **Figure 2A** shows a good clustering feature of the remaining 876 samples with clinical information of the patients, after removing 34 outlier samples. The co-expression network was then constructed with the WGCNA package in the R software. The soft‐threshold (power = 12) was determined based on the maximal R^2^ for the scale‐free network, which also had a relatively high Mean Connectivity (**Figure 2B**). To evaluate the distance between each gene pair, the topological overlap measure (TOM) matrix similarity was adopted. Hierarchical clustering analysis with the average method and dynamic method was used to build the cluster tree and classify the genes into modules, respectively (**Figure 2C**). After merging small modules according to their distances, 18 modules, with sizes ranging from 75 to 6628 genes, were constructed and randomly assigned with corresponding artificial colors (**Figure S2A**). The clustering of the modules and their correlations based on the module eigengenes are presented in **Figure 2D**.

### Correlation Test With Clinical Data Uncovered Statin-Related Co-expression Modules

**Figure 3:**
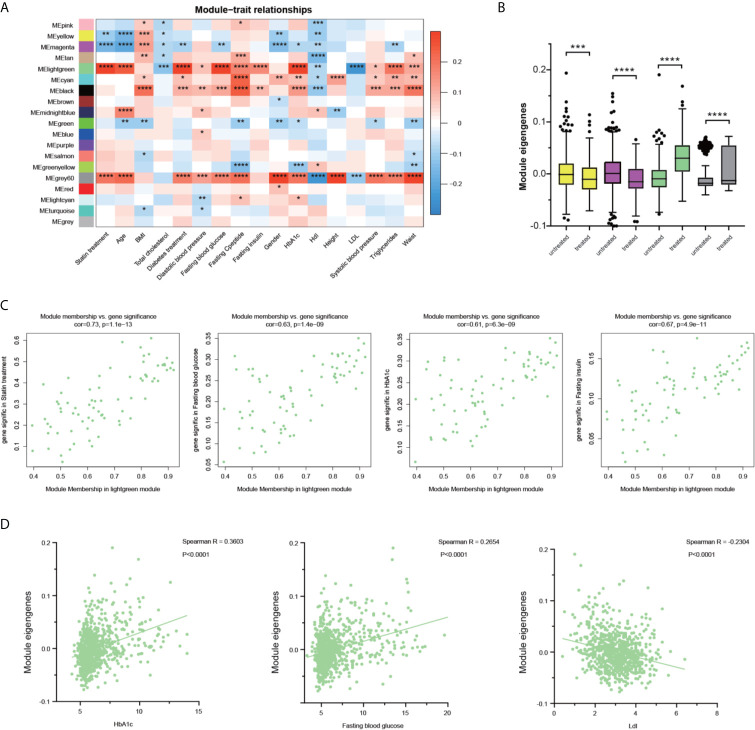
Identification of the key modules associated with glucose and lipid disorder after statin treatment. **(A)** Correlation between modules and clinical parameters. The significance of each correlation was marked and the color reflects the correlation coefficient. **(B)** The differences of the module eigengenes of each module between statin and non-statin treated patients. The color indicated the corresponding module and Wilcoxon signed-rank test were applied for the data that was not normally distributed. **(C)** Scatter plot for correlation between gene module membership in the light green module and the gene significance of the indicated parameters. **(D)** Scatter plot for correlation between patient green module eigengene and indicated parameters. Data were expressed as mean ± SD. *P < 0.05, **P < 0.01, ***P < 0.001, ****P < 0.0001.

To investigate the association of statin-related clinical futures and the WGCNA modules, the correlation coefficients and corresponding p‐values between patients’ clinical parameters and the eigengenes of each module were calculated. As shown in **Figure 3A**, multiple modules showed a significant though moderate correlation with patients’ clinic signatures. The yellow and magenta modules showed a similar behavior of negative correlation with statin treatment, but no significant correlations with metabolic parameters were judged by fasting blood glucose and HbA1c. The modules of black, grey60, and light green showed a possible correlation with multiple blood glucose parameters and a negative correlation with cholesterol metabolism. However, the black module did not show a correlation with statin treatment. The comparison of the eigengenes of all 18 modules (**Figure S2B**) confirmed the above findings of four modules that exhibited significant differences regarding statin treatment conditions (**Figure 3B**). Together, in both analyses, the light green module showed the strongest correlation of statin treatment with lower total cholesterol and Ldl, and higher glucose metabolism, and was thus chosen for further investigation.

We then correlated the module membership of each gene, indicating its hierarchy and connections with other members, through its clinical readouts. The scatterplots of “The Gene Significance *vs.* Module Membership” of each gene in the light green module with statin treatment, HbA1c, fasting blood glucose, fasting insulin are plotted respectively in **Figure 3C**. There are strong correlations between Gene Significance and Module Membership, indicating that the genes with higher connections with their module members also had a tighter correlation with glucose disorder phenotypes. It is noteworthy that in individual patients, the module eigengenes were positively correlated with HbA1c and fasting blood glucose, and negatively correlated with Ldl levels (**Figure 3D**). Additional parameters, including the age, BMI, total cholesterol, and diastolic blood pressure were also studied and the strong correlation of hub genes in the light green module with age and total cholesterol, and a moderate correlation with diastolic blood pressure were observed (**Figure S8**).

### Functional Enrichment of Light Green Genes Indicated the Key Network Altered Upon Statin Treatment in the Liver

**Figure 4:**
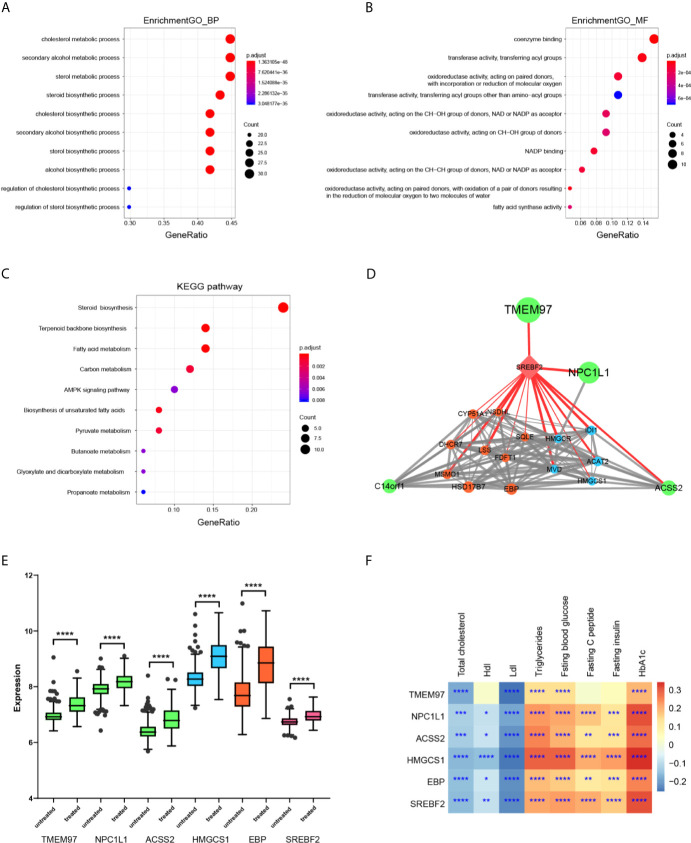
Functional enrichment and gene network construction of light green genes. **(A)** The top 10 biology process GO terms in the enrichment analysis of the light green genes. **(B)** The top 10 molecular function GO terms in the enrichment analysis of the light green genes. **(C)** The top 10 KEGG pathways in the enrichment analysis of the light green genes. **(D)** Protein-protein interaction (PPI) network of genes with a weight value > 0.8 in the light green module was constructed according to biological association. The nod size was mapped according to the average shortest path and the width of the edges was mapped according to the combined score, indicating the strength. The color indicates the pathway involved in each gene. **(E)** The differences of indicated genes between statin and non-statin treated patients. The color indicates that the corresponding pathway of the gene and the Wilcoxon signed-rank test was applied for the data, but it was not normally distributed. **(F)** Heatmap of the correlation of indicated genes with metabolic parameters. Data were expressed as mean ± SD. *P < 0.05, **P < 0.01, ***P < 0.001, ****P < 0.0001.

To explore the biological functions of the light green module, Gene Ontology (GO) biological process enrichment analysis was performed using the clusterProfiler R package. There was significant enrichment of GO terms in the biology processes of the cholesterol biosynthetic process (GO:0006695), secondary alcohol biosynthetic process (GO:1902653), and sterol biosynthetic process (GO:0016126) (**Figure 4A**). there was enrichment in molecular function terms of coenzyme binding (GO:0050662), transferase activity, transferring acyl groups (GO:0016746), oxidoreductase activity, acting on paired donors, with incorporation or reduction of molecular oxygen (GO:0016705) (**Figure 4B**). Searching for KEGG pathway mapping showed that the pathways of Steroid biosynthesis (hsa00100), Terpenoid backbone biosynthesis (hsa00900), and Fatty acid metabolism (hsa01212) were enriched in the light green module (**Figure 4C**). The genes in the string of synthesis of cholesterol, campesterol, brassicasterol, and stigmasterol were significantly enhanced (**Figure S3**).

Since highly connected hub nodes at the center of the network’s architecture are more likely to be the key drivers to respective cellular functions, the gene correlation network based on TOM of the light green genes was constructed. The genes that have a high Module Membership (>0.8) in the light green module showed tight correlations with each other, suggesting the existence of a functional core (**Figure S4A**). These genes were then submitted to the String database to construct the biological PPI network. As expected, the genes formed a highly connected network with the transcription factor SREBF2 as a hub gene transcriptionally controlling both steroid biosynthesis and terpenoid backbone biosynthesis genes that are the major component enzymes of the mevalonate pathway downstream to HMG-CoA reductase. Interestingly, SREBF2 also transcriptionally controls TEMEM97 and NPC1L1, which were suggested to be involved in cell signaling and cholesterol uptake (**Figure 4D**). In addition, ACSS2, an enzyme that synthesizes Acetyl-CoA from short-chain fatty acids was also identified as a core member of the network. Thus, the hub genes of the light green module were organized as a tightly connected functional unit involving cellular cholesterol uptake, Acetyl-CoA synthesizing as well as cholesterol and terpenoid syntheses in the liver. Finally, the feature genes at core position were validated between statin treated and non-treated patients (**Figure 4E**). As shown, all the genes were significantly higher in statin treated patients (the full profile of light green genes was shown in **Figure S5**) and they all showed the same pattern as the light green module, which was remarkably correlated with glucose disorder parameters while negatively correlated with Ldl (**Figure 4F** and **Figure S6**).

### Statin Increased Protein Acetylation in Hepatocytes

**Figure 5:**
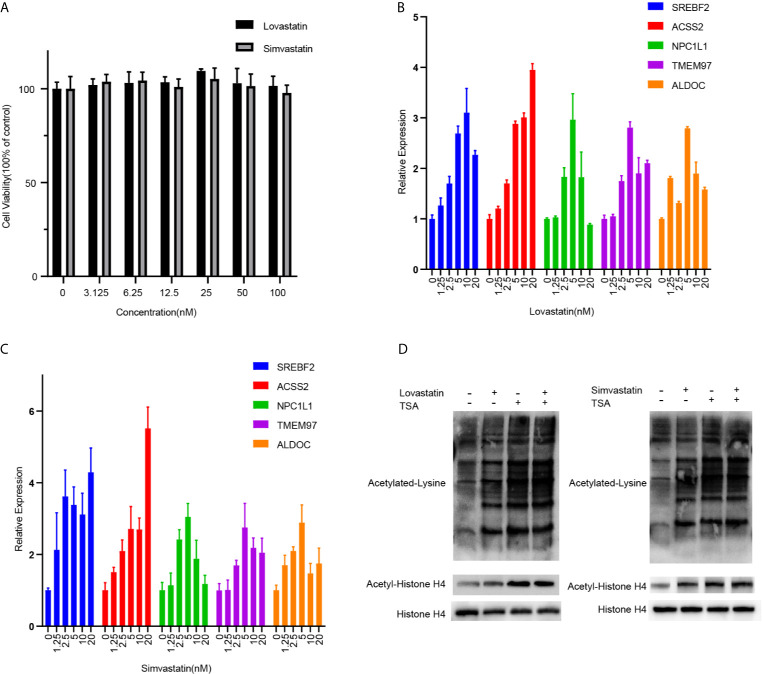
QPCR validation and analysis of protein acetylation in hepatocytes. **(A)** LO2 cells were treated with lovastatin or simvastatin for 24 hours. The cell viability was analyzed by MTS mothed. The mRNA expressions of indicated genes in LO2 cells after treated with lovastatin **(B)** or simvastatin **(C)** for 24 hours were tested by real-time quantitative PCR. **(D)** Western blot analyses of Acetylated lysine, Acetyl-histone H4, and total histone H4 in the LO2 cells after being treated with lovastatin, simvastatin, and TSA for 24 hours. Data were expressed as mean ± SD.

Next, we validated the transcriptions of key light green genes in hepatocytes. A human hepatocyte cell line (LO2) was treated with lovastatin or simvastatin. The treatment of both satins showed no obvious inhibition of the cell growth at indicated concentrations (**Figure 5A**) suggesting low cytotoxicity to hepatocytes. Consistent with the transcriptome data, the transcription levels of the key gene SREBF2, ACSS2, NPC1L1, and ALDOC et al. were all upregulated after statin treatment in the cells in a dose-dependent manner (**Figures 5B, C**). ALDOC, the gene that encodes the Fructose-1,6-Biphosphate Triosephosphate Lyase, was not in the biological annotated network, but has a high membership score. It was indeed upregulated after statin treatment and the only glucose metabolism-related gene identified in the expression hub. Furthermore, two key genes, ACSS2 and ACLY, which are critical for the synthesis of Acetyl-CoA, were also upregulated (**Figures 5B, C** and **Figure S5**), suggesting that the protein acetylation in the hepatocytes treated with statin might be altered, which may fundamentally affect cellular metabolism status (14). Statin moderately induced acetylation of pan-protein and histone H4 in the cells, compared with TSA treatment (**Figure 5D** and **Figure S9**).

## Discussions

A non-bias WGCNA analysis was performed to analyze the overall internal organization and connections of the genes in the transcriptome and to connect the gene modules with the clinical parameters in overweight patients after statin treatment. One critical module, the light green module, containing 75 genes that have a tight correlation with multiple glucose and lipid metabolic parameters, was uncovered. Unlike the DEG findings, the overall module organization of the transcriptome and the connections of the genes in the target module were assessed (**Figure S7**). Key genes were validated by QPCR in the hepatocytes upon statin treatment. Although their expressions were significantly altered in the hepatocytes, the underlying molecular function and influence on metabolic diseases still required further investigation.

Functional enrichment analysis based on the gene annotation of the light green module revealed a hierarchical network of 21 genes. These formed a tightly connected hub with functionally close relation to the core, which was largely transcriptionally controlled by SREBF2, but not SREBF1, in which most of the members are enzymes of the mevalonate pathway downstream to HMG-CoA reductase, the target of statin, and participate Steroid and Terpenoid backbone biosyntheses, suggest a counter reaction of the liver as the consequence of a low detecting level of cellular/plasma cholesterol resulting from statin treatment (Ldl from 3.12 to 2.64 mmol/l, p= 5.639e-09, total cholesterol from 5.00 to 4.65 mmol/l, p= 0.0002258). This robust reaction across the livers of the statin treated patients indicated that the cholesterol sensing system was likely compromised in overweight/high blood cholesterol patients, although a negative feedback mechanism to suppress the cholesterol synthesis genes was triggered, but at a higher Ldl level. Notably, at a reduced stringency of module membership, a secondary network involving Fatty acid metabolism genes was also identified along with an increase of the blood triglycerol levels in the patients who received statin treatment (**Figure S1** and **Figure S4B**). Whether this observation is due to an increase of the cytoplasmic Acetyl-CoA levels resulting from the inhibition of the HMGCR function has not yet been tested and is controversial (15), and further investigation is warranted.

SREBF2 is a well-known key transcriptional regulator in cholesterol biosynthesis that is sensitive to low levels of cholesterol. The generation of transcriptionally active SREBF2 is exquisitely dependent upon the content of cholesterol in the endoplasmic reticulum, which at transcription level auto regulates itself, and thus behaves as a cholesterol sensor and the organizer of cholesterol synthesis (16).

TMEM97, which is transcriptionally controlled by SREBF2, is an intracellular orphan receptor that binds numerous drugs and highly expressed in various proliferating cancer cells involving cell survival, morphology, and differentiation (17). It is wide phyletic distributed in eukaryotes (in plants, metazoa, and fungi) and likely to have a fundamental cellular function, but is poorly characterized (17). It was reported to regulate cellular cholesterol homeostasis and has a function of sterol isomerase (18). Under sterol-depleted conditions, it localizes to endo-/lysosomal compartments and binds to LDL cholesterol transport-regulating protein Niemann-Pick C1 (NPC1) (19). Whether TMEM97 in the liver may involve the regulation of glucose is still an open question. Moreover, in this study, an NPC1 like protein-1 (NPC1L1) was enriched and participated in the core network. It is critical for the uptake of cholesterol across the plasma membrane of the intestinal enterocyte (20). Naturally occurring mutations that disrupt NPC1L1 functions were found to be associated with reduced plasma LDL cholesterol levels and a reduced risk of coronary heart disease (21). The correlation with TMEM97 revealed in this study suggested that they play a role in regulating cholesterol uptake in the liver.

ACSS2 is an enzyme that synthesizes Acetyl-CoA from short-chain fatty acids (15). It may promote acetate utilization and maintains cancer cell growth under metabolic stress, acting as an energy sensor (22). It may also control acetate uptake and contribute to fatty acids and under the regulation of SREBF2 (23). Acetyl-CoA is the metabolite central to cholesterol synthesis, fatty acid synthesis, isoprenoid synthesis, and protein/histone acetylation, etc. (24, 25). Notably, another enzyme, ACLY, which catabolizes Acetyl-CoA from citrate was also significantly upregulated in the patients who received statin. Thus, the levels of Acetyl-CoA were likely enriched in the cells due to the upregulation of lipid importer as well as various ways of Acetyl-CoA biosynthesis, and the inhibition of the utilization of it to synthesize steroids with the presence of statin. The hyper acetylation of proteins may fundamentally affect cellular metabolism (26).

Interestingly, the current study did enrich a fructose metabolic gene, ALDOC, which has been implicated in obesity and type II diabetes (27). Whether the increased expression of this gene is involved in a higher level of blood glucose in statin treated patients and the potential mechanism are currently not known. Moreover, a manual inspection of genes involving gluconeogenesis, glycogenolysis, as well as lipid transporters such as CD36 and FABPs, etc. did not observe significant upregulation. Thus, the current analysis of transcriptome in the livers of statin treated patients did not provide direct information on statin-caused glucose metabolic disorders. However, the literature did indicate the harmful effect of statin on β cells and muscles (5, 28), which is beyond the scope of the current study.

## Conclusion

In this study, the transcriptome of the livers of obesity and hypercholesterolemia patients was re-analyzed by Weighted gene co-expression network analysis. A multiple module organization was revealed that was related to various clinical metabolic parameters. Specifically, a lipogenic expression hierarchical network involving both cholesterol and fatty acid syntheses in the liver of statin treated patients was identified, which is centered by SREBF2 and highly coexpressed at the transcription level. QPCR validated the consistency of the key genes of the network in the hepatocytes upon statin treatment. The upregulation of the key enzymes involved in the synthesis of Acetyl-CoA induced gentle global acetylation of pan-protein in hepatocytes and acetylation of histone H4. Overall, this study provides insight into the effect of statin on transcriptional and post-transcriptional regulation of genes in the liver. 

## Data Availability Statement

The raw data supporting the conclusions of this article will be made available by the authors, without undue reservation.

## Author Contributions

SS, HW, and QG designed the work and wrote the manuscript. MN, QL, and LW retrieved and analyzed the transcriptome data. SS and HM performed the QPCR and WB assays. YP prepared the figures. All authors read and approved the final manuscript.

## Funding

This work was supported by grants from the National Key R&D Program of China (2020YFC2005600/01), the Fundamental Research Funds for the Central Universities (0214-14380509), the National Key R&D Program of China (2018YFC2001800, 2020YFC2005100, 2020YFC2005300), the Science and Technology Program of Karamay City (2018HM014A), the National Natural Science Foundation of China (No. 82070912), the Key Project of Research and Development of Ningxia Hui Autonomous Region of China (No. 2017BN04), and a grant from the Natural Science Foundation of Jiangsu Province China (No. BK20171347 and BE2019676).

## Conflict of Interest

The authors declare that the research was conducted in the absence of any commercial or financial relationships that could be construed as a potential conflict of interest.

## Supplementary Material

Figure S1 Boxplot of clinical parameters between statin treated and non-treated patients. *P < 0.05, ****P < 0.0001, ns no significant.

Figure S2
**(A)** Barplot of counts of genes of each module. The counts were log transformed. **(B)** Boxplot of module eigengenes between statin treated and non-treated patients. ***P < 0.001, ****P < 0.0001.

Figure S3 KEGG pathways of **(A)** steroid biosynthesis, **(B)** terpenoid backbone biosynthesis **(C)** free fatty metabolism. Genes in the light green module are marked red.

Figure S4
**(A)** Interaction network of high membership genes in the light green module according to TOM. The size of the nod was mapped to its membership score. **(B)** Interaction network of genes in the light green module involving fatty acid metabolism according to string interaction. The size of the nod was mapped to the number of directly interacting nods. The width of the edge was mapped to the combined score.

Figure S5 Boxplot of the expression of the genes in the light green module between statin treated and none treated patients. Except for “ns” marked genes, all other genes showed a significant difference.

Figure S6 Heatmap of the correlation of the genes in the light green module with indicated clinical parameters. Genes shown in **Figure 4F** are marked with *.

Figure S7
**(A)** Correlation matrix according to Spearman's rank correlation coefficient of top 25 DEGs between statin treated and non-treated patients. **(B)** Correlation matrix according to Spearman's rank correlation coefficient of all the light green genes.

Figure S8 Scatter plot for correlation between patient green module eigengene and indicated parameters.

Figure S9 Coomassie Brilliant Blue staining of the total protein of LO2 cells receiving lovastatin, simvastatin, and TSA treatment.
