# Supplementary figures and images for: Statin Treatment Induced a Lipogenic Expression Hierarchical Network Centered by SREBF2 in the Liver
Source: Front Endocrinol (Lausanne). 2021 Jul 19;12:573824. doi: 10.3389/fendo.2021.573824 (PMC8326809; doi:10.3389/fendo.2021.573824)

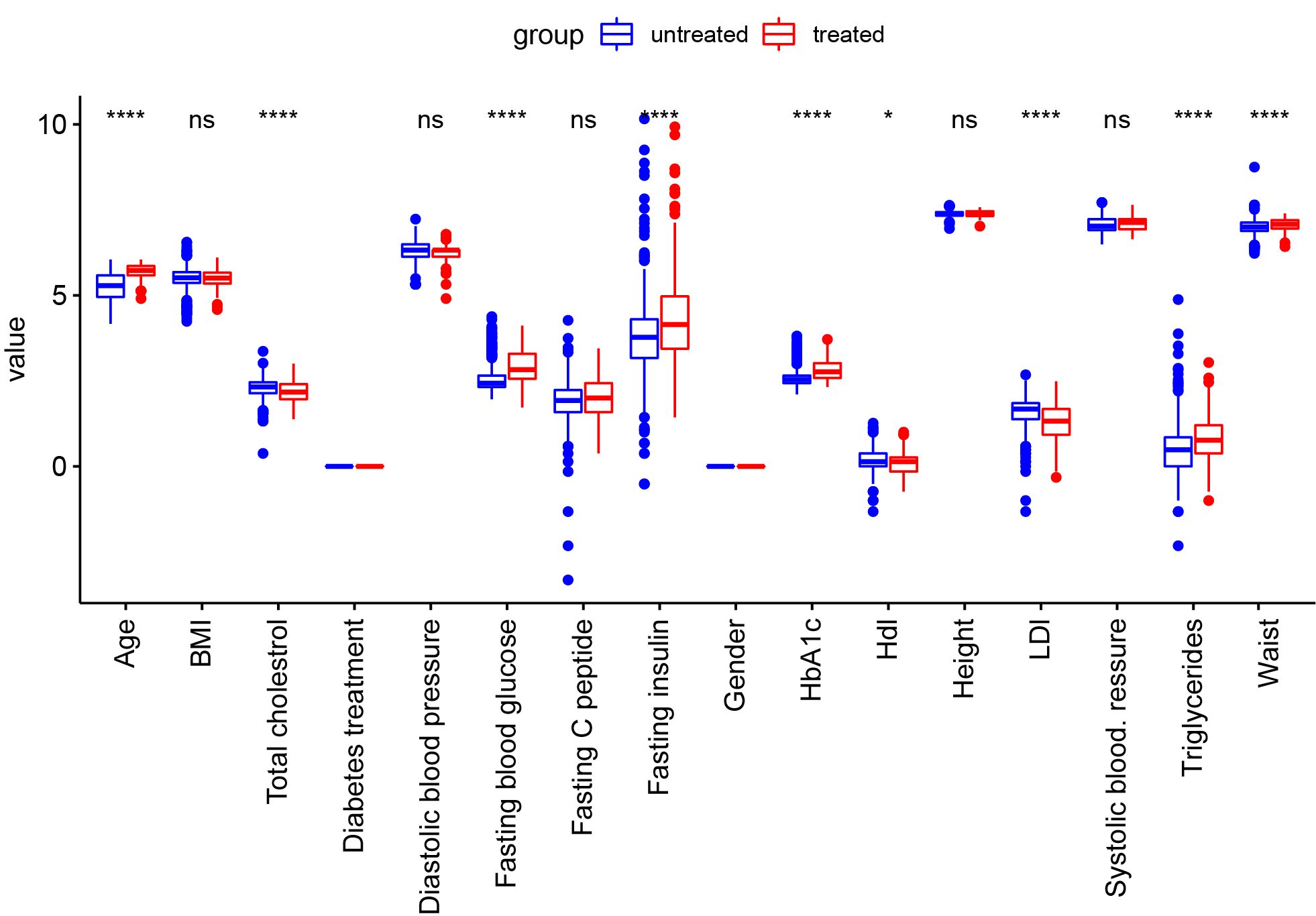

Supplement: Figure S1 — Boxplot of clinical parameters between statin treated and non-treated patients. *P < 0.05, ****P < 0.0001, ns no significant. [file Image_1.tif]

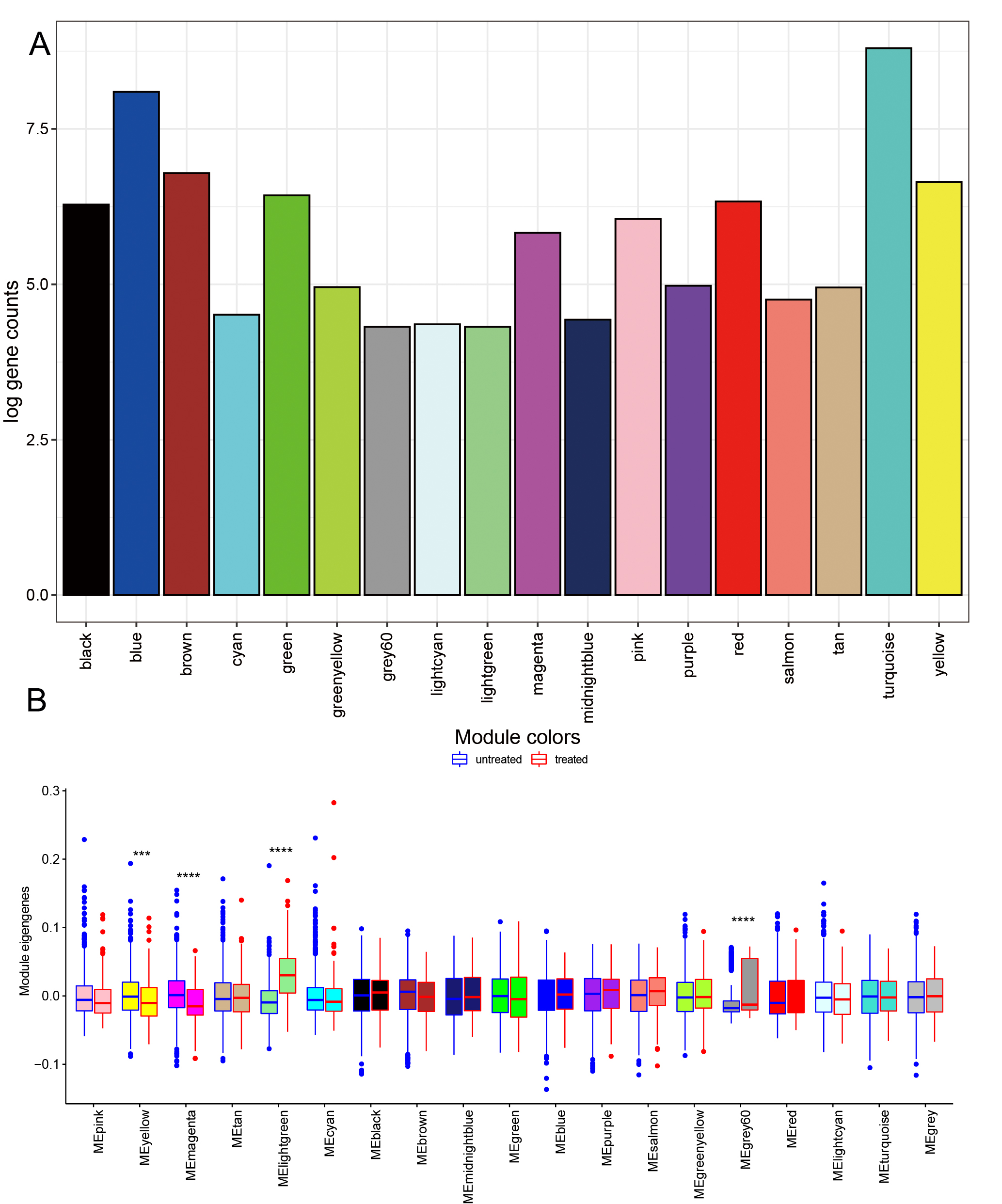

Supplement: Figure S2 — (A) Barplot of counts of genes of each module. The counts were log transformed. (B) Boxplot of module eigengenes between statin treated and non-treated patients. ***P < 0.001, ****P < 0.0001. [file Image_2.tif]

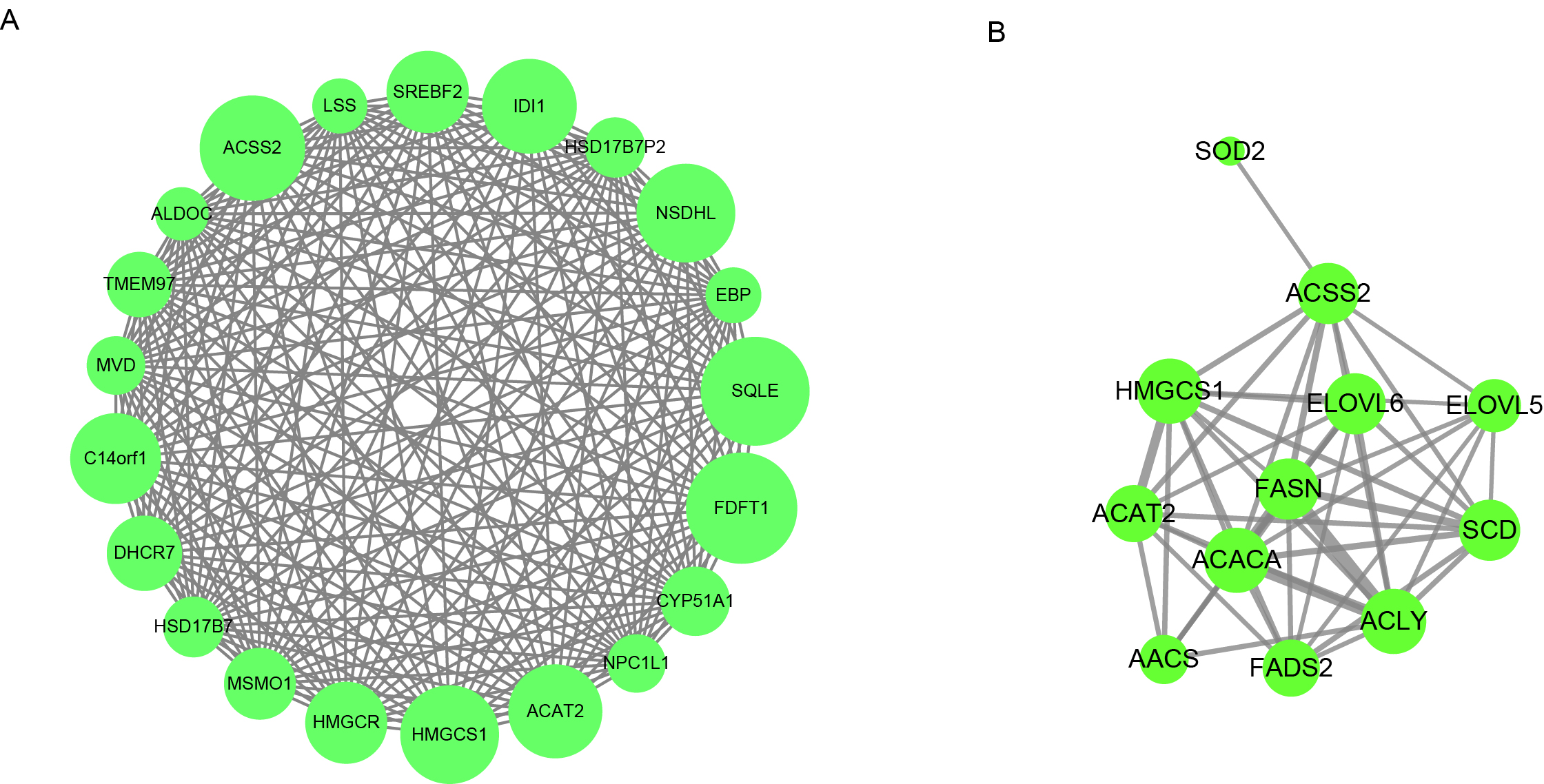

Supplement: Figure S4 — (A) Interaction network of high membership genes in the light green module according to TOM. The size of the nod was mapped to its membership score. (B) Interaction network of genes in the light green module involving fatty acid metabolism according to string interaction. The size of the nod was mapped to the number of directly interacting nods. The width of the edge was mapped to the combined score. [file Image_4.tif]

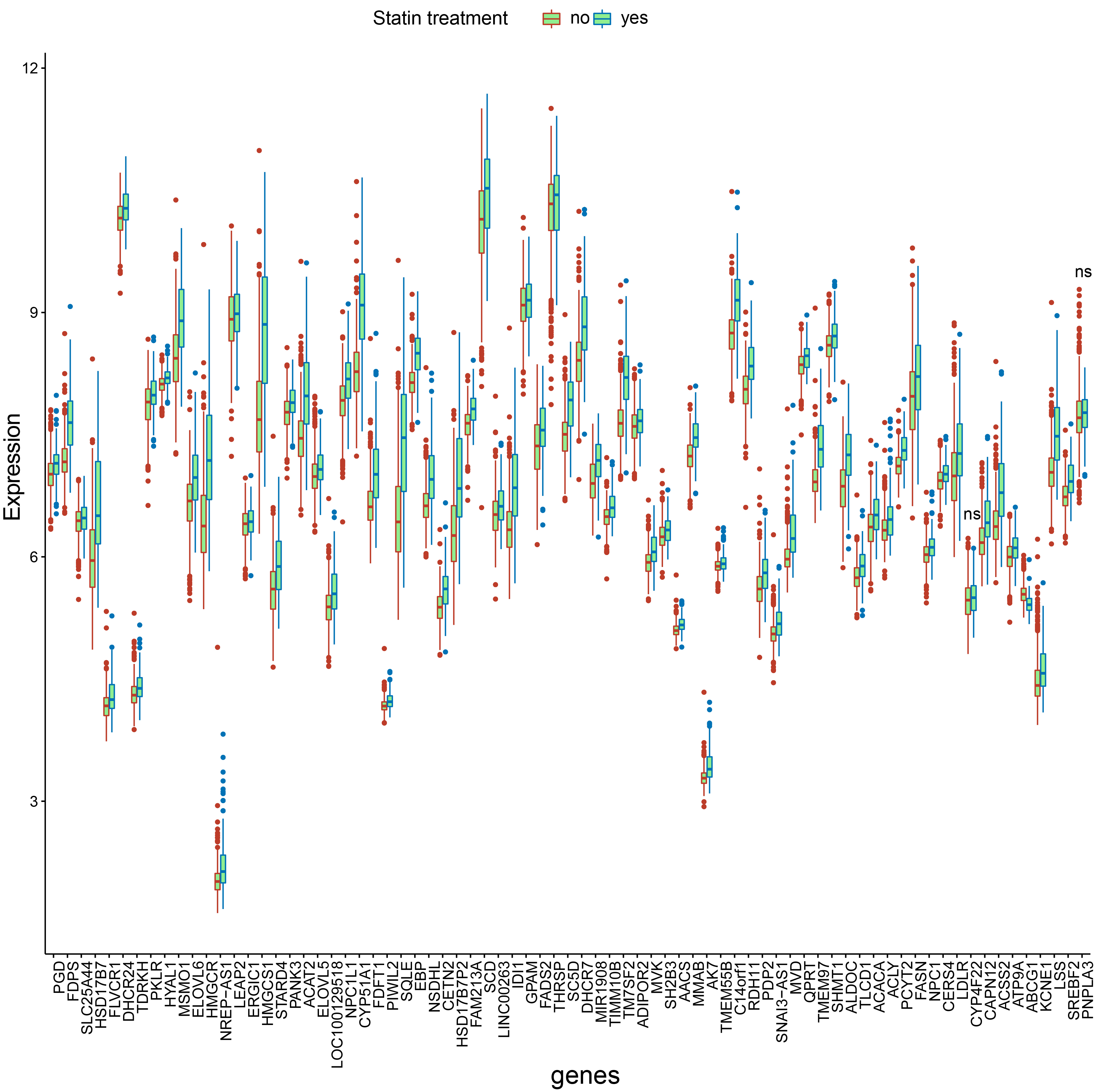

Supplement: Figure S5 — Boxplot of the expression of the genes in the light green module between statin treated and none treated patients. Except for “ns” marked genes, all other genes showed a significant difference. [file Image_5.tif]

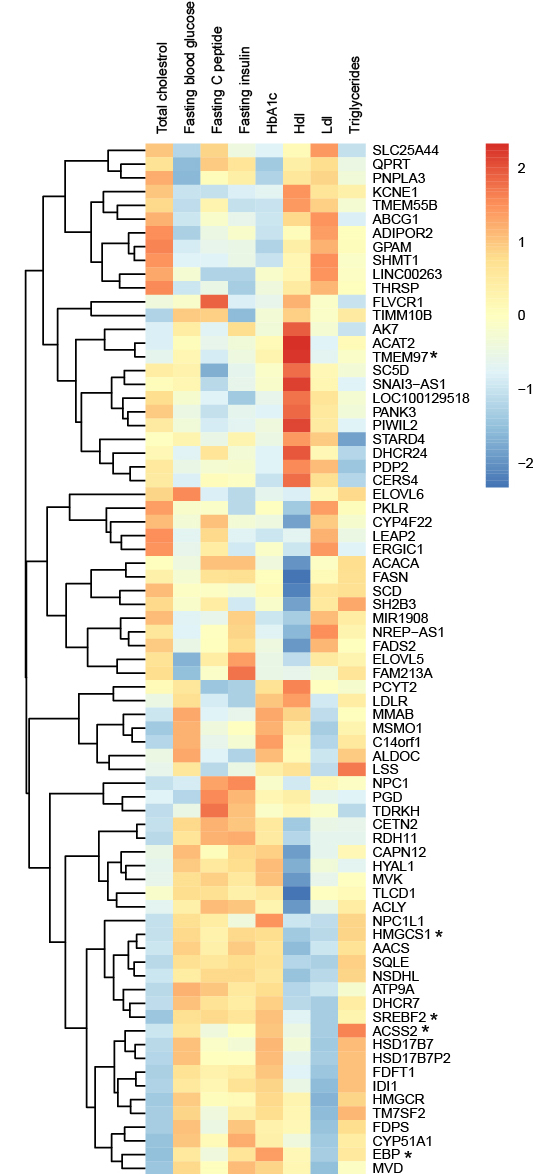

Supplement: Figure S6 — Heatmap of the correlation of the genes in the light green module with indicated clinical parameters. Genes shown in Figure 4F are marked with *. [file Image_6.tif]

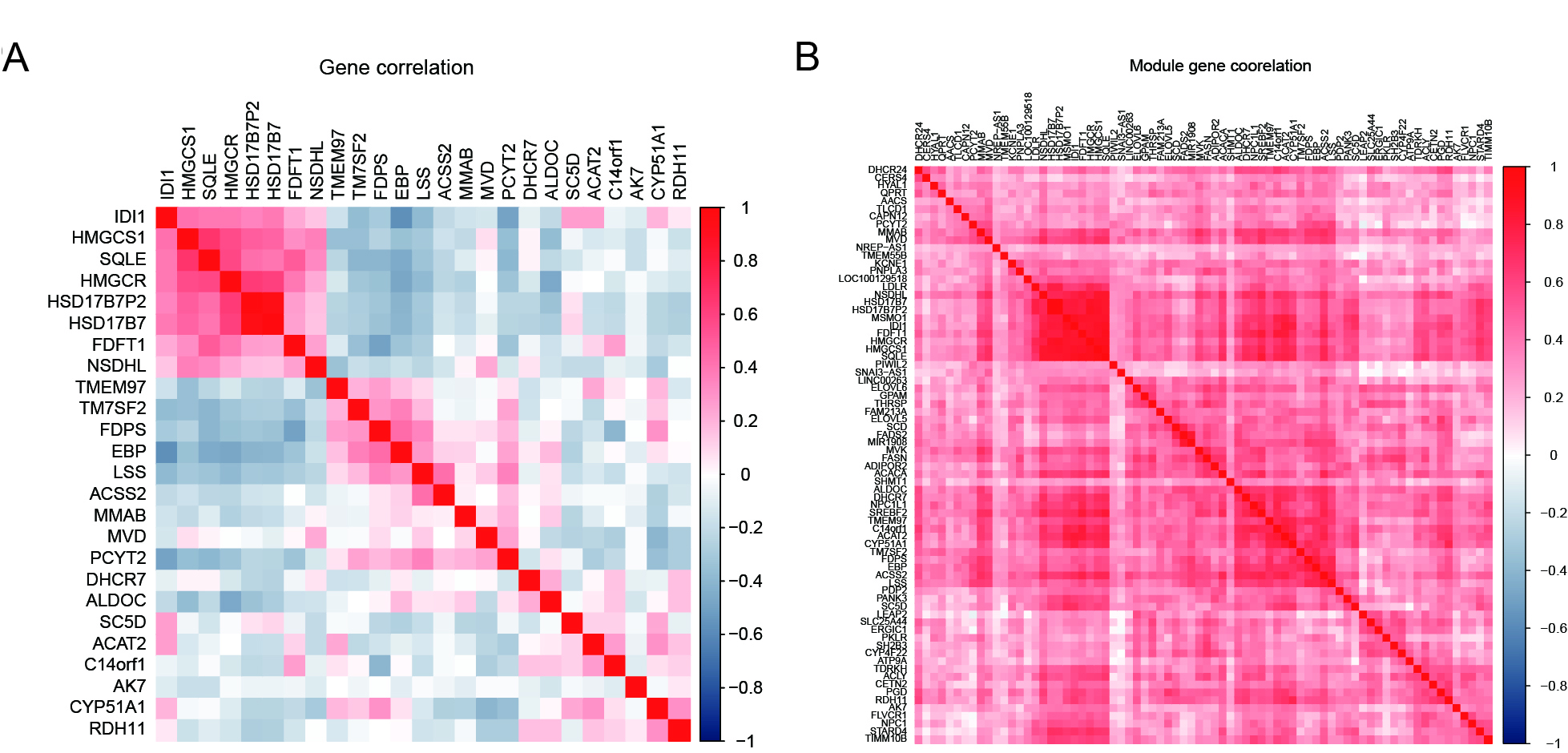

Supplement: Figure S7 — (A) Correlation matrix according to Spearman's rank correlation coefficient of top 25 DEGs between statin treated and non-treated patients. (B) Correlation matrix according to Spearman's rank correlation coefficient of all the light green genes. [file Image_7.tif]

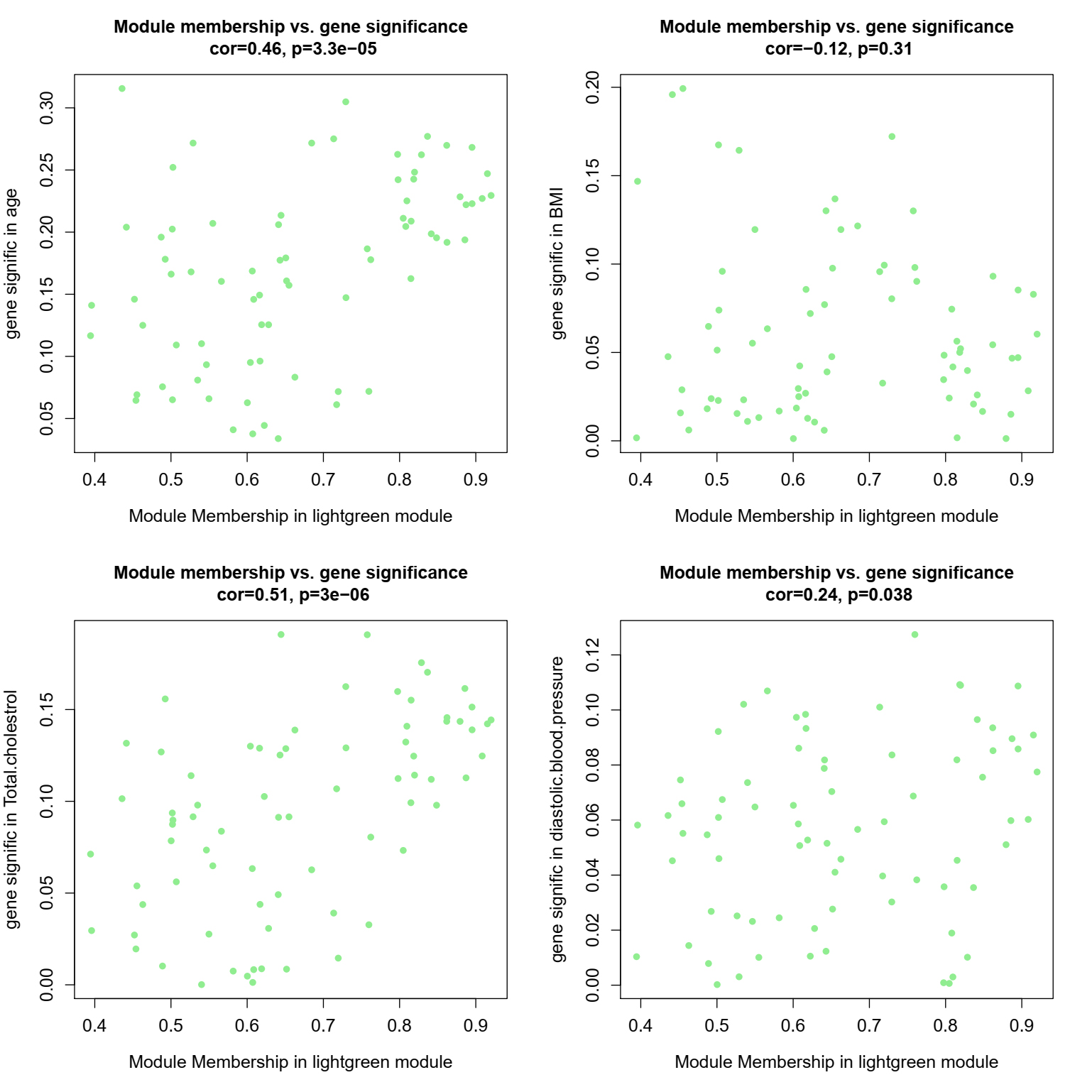

Supplement: Figure S8 — Scatter plot for correlation between patient green module eigengene and indicated parameters. [file Image_8.tif]

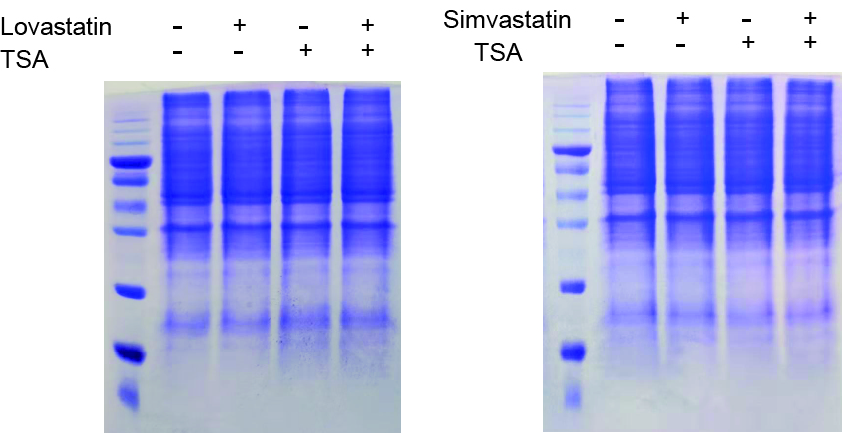

Supplement: Figure S9 — Coomassie Brilliant Blue staining of the total protein of LO2 cells receiving lovastatin, simvastatin, and TSA treatment. [file Image_9.tif]
