# Supplementary material for: Statin Treatment Induced a Lipogenic Expression Hierarchical Network Centered by SREBF2 in the Liver
Source: Front Endocrinol (Lausanne). 2021 Jul 19;12:573824. doi: 10.3389/fendo.2021.573824 (PMC8326809; doi:10.3389/fendo.2021.573824)
Supplement: Supplementary file 10 [file Table_1.docx]

| Target | Sequence |
| --- | --- |
| SREBF2-Forward | 5'-GGAGACCATGGAGACCCTCA-3' |
| SREBF2-Reverse | 5'-GTCAGGGAACTCTCCCACTTG-3' |
| TMEM97-Forward | 5'-GCGAGCTCTACCCAGTCG-3' |
| TMEM97-Reverse | 5'-GACTTAAACCAGGCTGGGGG-3' |
| NPC1L1-Forward | 5'-CCAAGTCGACTGGAAGGACC-3' |
| NPC1L1-Reverse | 5'-TAGTCAGCCATGCAGCTCAG-3' |
| ACSS2-Forward | 5'-GGAGTTGGTCTCCGCCG-3' |
| ACSS2-Reverse | 5'-TGGCAATGTCTCCCCAGAAT-3' |
| ALDOC-Forward | 5'-CTGCAGCCTCATCTGTTTGC-3' |
| ALDOC-Reverse | 5'-CAGAAAGGGCTGGGTACGAG-3' |
